# Adipo-oncology: adipocyte-derived factors govern engraftment, survival, and progression of metastatic cancers

**DOI:** 10.1186/s12964-024-01474-4

**Published:** 2024-01-18

**Authors:** Shinya Sato

**Affiliations:** 1 Morphological Analysis Laboratory, Kanagawa Cancer Center Research Institute, 2-3-2, Asahi-Ku, Yokohama, Kanagawa 241-8515 Japan; 2 Molecular Pathology and Genetics Division, Kanagawa Cancer Center Research Institute, 2-3-2, Asahi-Ku, Yokohama, Kanagawa 241-8515 Japan; 3 Department of Pathology, Kanagawa Cancer Center Hospital, 2-3-2, Asahi-Ku, Yokohama, Kanagawa 241-8515 Japan

**Keywords:** Adipocyte, Adipokines, Adipo-oncology, Tumor metastasis, Tumor microenvironment

## Abstract

Conventional therapies for metastatic cancers have limited efficacy. Recently, cancer therapies targeting noncancerous cells in tumor microenvironments have shown improved clinical outcomes in patients. However, further advances in our understanding of the metastatic tumor microenvironment are required to improve treatment outcomes. Adipocytes are distributed throughout the body, and as a part of the metastatic tumor microenvironment, they interact with cancer cells in almost all organs. Adipocytes secrete various factors that are reported to exert clinical effects on cancer progression, including engraftment, survival, and expansion at the metastatic sites. However, only a few studies have comprehensively examined their impact on cancer cells. In this review, we examined the impact of adipocytes on cancer by describing the adipocyte-secreted factors that are involved in controlling metastatic cancer, focusing on adipokines, such as adiponectin, leptin, visfatin, chemerin, resistin, apelin, and omentin. Adipocyte-secreted factors promote cancer metastasis and contribute to various biological functions of cancer cells, including migration, invasion, proliferation, immune evasion, and drug resistance at the metastatic sites. We propose the establishment and expansion of “adipo-oncology” as a research field to enhance the comprehensive understanding of the role of adipocytes in metastatic cancers and the development of more robust metastatic cancer treatments.

## Introduction

With the aging of society, the incidence of cancer is increasing worldwide, and cancer has become the leading cause of death, particularly in developed countries [1, 2]. To date, cancer therapies, including surgery, anticancer drugs, and radiation, have targeted cancer cells and have been effective to a certain extent. However, the effectiveness of conventional therapies is limited, particularly in cases of metastatic cancers.

In recent years, cancer therapies that target noncancerous cells within tumor microenvironments have emerged. Of these, the best-known are therapies targeting immune cells, such as immune checkpoint inhibitor-based therapies, which have markedly improved the outcomes of certain aggressive cancers, including melanoma [3–6]. Therapies targeting blood vessels are also becoming more common, and vascular-targeted therapies have been incorporated into the standard treatment regimens for some cancers [7–9]. Recently, cancer-associated fibroblasts (CAFs), another major host cell type, have been studied for targeted therapies [10]. However, even with the application of these non-neoplastic cell-targeting therapies, a complete cure for all cancers is yet to be achieved. This is partly because our understanding of the microenvironment surrounding cancer cells is incomplete. Despite the wide variety of cell types that comprise the metastatic tumor microenvironment, the function of only few of the resident cells have been examined in terms of cancer progression. Therefore, a better understanding of the metastatic tumor microenvironment is required.

Adipocytes are a significant component of the metastatic tumor microenvironment. They are distributed throughout the body and become part of the metastatic tumor microenvironment by interacting with cancer cells in almost all organs, except the brain. Adipocytes secrete various factors, including cytokines (adipokines), proteases, chemokines, lipokines, vasoactive factors, coagulation regulators, free fatty acids, amino acids, steroids, nucleotides, and extracellular vesicles [11–14]. Many of these secretory factors exert clinical effects on cancer progression according to previous studies [15–18]. Although adipocytes are a thus critical factors for understanding the metastatic tumor microenvironment, few studies have comprehensively examined their impact on cancer cells.

We have recently uncovered various roles of adipocytes in cancer progression [18], based on analyses of histopathology, CAF induction, immune evasion, proliferation, and dormancy. In addition, we are actively conducting in vitro, in vivo, and translational studies.

**Fig. 1:**
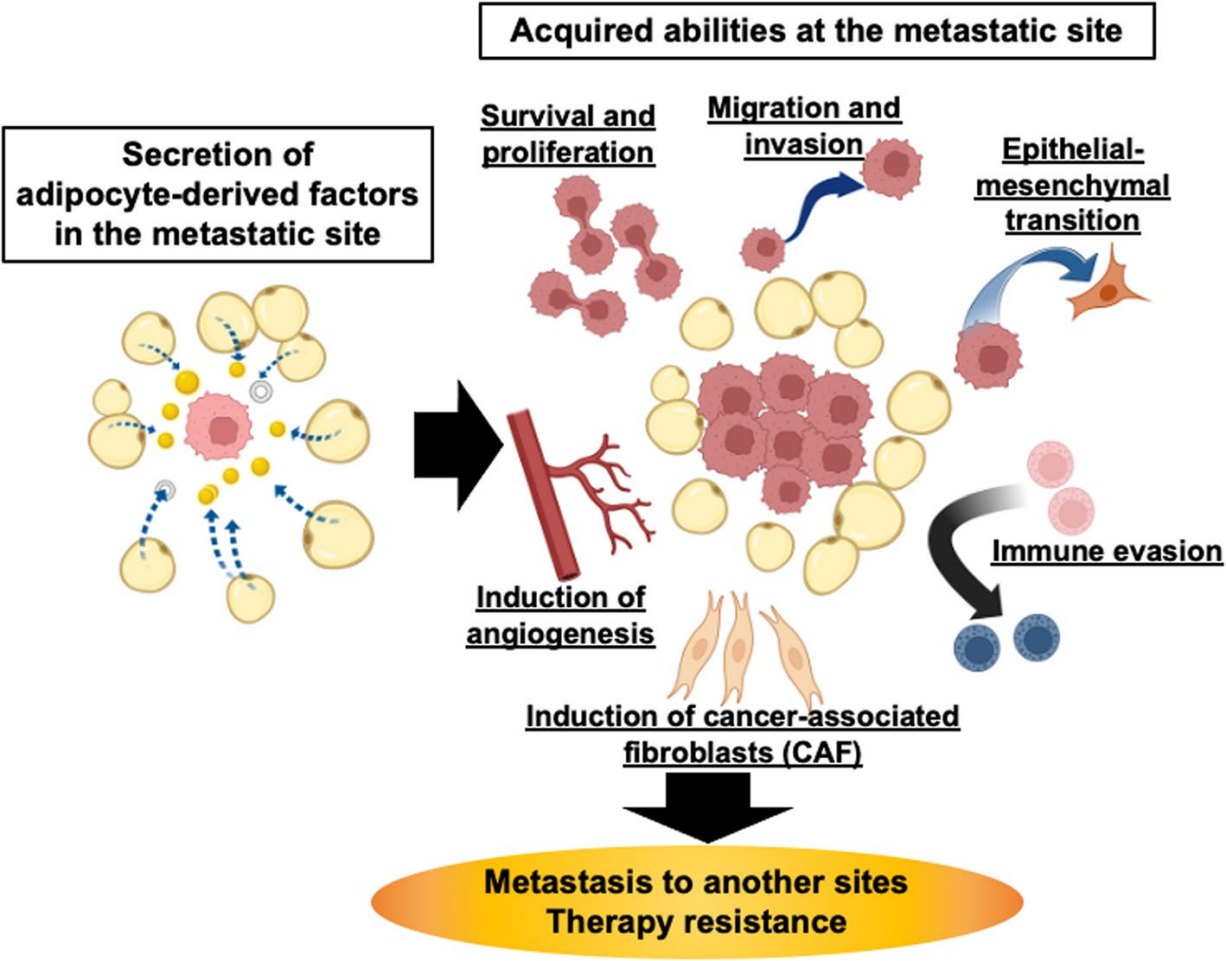
Adipocyte-derived factors govern engraftment, survival, and progression of metastatic cancers

In this review, we systematically examined the impact of adipocytes on metastatic cancer by describing the effect of the factors they secrete on cancer, with a particular focus on adipokines, which are mainly produced by adipocytes (Fig. 1, Table 1).

**Table 1:** Receptors, effects on cancer cells, and activation of signaling pathways by adipokines

Name	Receptors	Effects of adipokines on tumor progression	Activating signaling pathways	Ref
**Adiponectin**	AdipoR1 AdipoR2	Adiponectin basically suppresses cell proliferation, migration, invasion, and metastasis of cancers. Adiponectin promotes angiogenesis in breast cancer and other cancers. Adiponectin level correlates with cancer-related cachexia	AMPK p38 MAPK PI3K/AKT WNT	[19–34]
**Apelin**	Apelin receptor/ APJ/APLNR	Apelin promotes proliferation, migration, and invasion of cancer cells. Apelin promotes angiogenesis	PI3K/AKT	[35–40]
**Chemerin**	CMKLR1 GPR1	Chemerin regulate proliferation, invasion and metastasis differently among cancers derived from different organs Chemerin strengthens immune system in breast cancer	PI3K/AKT	[41–49]
**Leptin**	Leptin receptors/ Ob-Rs	Leptin promotes proliferation, migration, invasion, metastasis, epithelial-mesenchymal transition, and drug resistance of cancer cells. Leptin induces cancer-associated fibroblasts and lymphangiogenesis	JAK/STAT SAPK/JNK MEK/ERK1/2 Notch PI3K/AKT	[50–71]
**Omentin**	Unknown	Effects of omentin on tumor progression is different depending on the primary sites of cancer or the patient’s health status	JNK	[72–77]
**Resistin**	CAP1 Decorin ROR1 TLR4	Resistin promotes proliferation, migration and invasion and epithelial-mesenchymal transition, and drug resistance of cancer cells. Resistin promotes angiogenesis	JAK/STAT PI3K/AKT WNT	[78–88]
**Visfatin**	Unknown	Visfatin promotes proliferation, invasion, and inhibition of apoptosis of cancer cells	p38 MAPK MEK/ERK1/2 PI3K/AKT	[89–95]

### Adiponectin

Adiponectin is a common adipokine. It was discovered in 1996 as the gene with the most abundant expression in adipose tissue [96]. Adiponectin is now known to be secreted by the muscles, brain, and other tissues, in addition to adipose tissue [96, 97]. The protein is a polypeptide composed of 244 amino acids, and it plays a vital role in glucose and fatty acid metabolism [98, 99]. High adiponectin levels decrease the risk of diabetes [100, 101]. Adiponectin secretion is stimulated by calorie restriction and exercise [102, 103].

Notably, white adipocytes secrete more adiponectins compared to beige or brown adipocytes [104, 105]. However, when white adipose tissues undergo browning due to drug administration, the amount of secreted adiponectin increases [106]. In contrast, knockout (KO) of Zfp423, which negatively regulates Prdm16 expression, tends to decrease adiponectin secretion in browned white adipocytes [107].

The adiponectin receptors ADIPOR1 and ADIPOR2 were first reported in 2003 [108], followed by the discovery of T-cadherin as another adiponectin receptor in 2004 [109]. These receptors are mainly distributed in the muscle tissue and vascular endothelium [110]. ADIPOR1 and ADIPOR2 have been reported to activate AMPK signaling and suppress MAPK, PI3K/AKT, and WNT signaling [108, 110, 111]. In contrast, T-cadherin, considered a non-signaling protein, has been reported to bind adiponectin and induce AMPK phosphorylation in the myocardium [112].

Adiponectin is mainly involved in the inhibition of tumor growth in nasopharyngeal [19], ovarian [20], hepatocellular [21], pancreatic [22], breast [23], colon [24], and prostate cancers [25] as well as in malignant mesotheliomas [26] and glioblastomas [27]. Tumor growth suppression mechanisms include activation of ADIPOR1 signaling, which induces AMPK phosphorylation, attenuation of the β-catenin signaling pathway [22], and activation of MAPK [23], ERK1/2, and AKT signaling [27]. In an in vivo study, adiponectin treatment suppressed transplanted colon tumor growth, and regulated metabolic, inflammatory and cell cycle signaling in colon cancer [28].

To date, adiponectin has not been directly administered to patients with cancer in any trials. Most cancer-related trials involving adiponectin use it as a marker of feasible output from weight loss or a healthy diet [29–32]. No direct correlation between adiponectin levels and clinical outcomes, such as cancer recurrence or metastasis, has been reported in randomized controlled trials to date. However, partial effects of serum adipocyte level on the density of tumor-infiltrating lymphocytes have been reported in patients with Stage III colon cancer [33]. Notably, abnormal levels of serum adiponectin are associated with shorter progression-free survival in metastatic colorectal cancer [34]. Furthermore, in a randomized clinical trial, a dose–response effect of exercise was observed to increase adiponectin levels and potentially reduce the risk of breast cancer [30].

### Leptin

Leptin, a member of the adipokine family of proteins, is secreted by adipocytes. Leptin maintains calorie consumption and is mainly involved in energy storage by storing of triglycerides in the adipose tissues [113, 114]. Leptin overexpression reduces triglyceride concentrations [115, 116]. The leptin receptor was first identified as OB-R in 1995 [117]. Leptin receptors have various isoforms [118], which differ in the length of their intracellular domains. OB-Rfl, which has the longest intracellular domain, can activate the JAK-STAT pathway [118, 119]. In contrast, short isoforms are reported to activate MAPK signaling pathways [120]. Soluble leptin receptors are also present, and their levels correlate with the number of membrane leptin receptors and have been reported to be increased by obesity [121, 122]. Leptin primarily regulates brain function and the levels of brain-derived hormones [123, 124]. Leptin receptors are typically distributed in the central nervous system [125, 126]. However, it is now known that leptin receptors are also distributed in liver cells (hepatocytes), adipocytes, fibroblasts, and endothelial cells [50, 127, 128]. Leptin has been reported to serve as a proliferative factor in tumors derived from multiple organs, such as the lungs [51, 52], liver [53], breast [54, 55], prostate [56, 57], pancreas [58], ovaries [59, 60], brain [61], and colorectum [62], exerting its effects via the JAK/STAT, MEK/ERK1/2, NOTCH, JNK, and/or PI3K/AKT signaling pathways. In an in vivo study, a leptin receptor antagonist prolonged the average survival time of a mouse xenograft model of triple-negative breast cancer cell lines [63]. In addition, overexpression of the leptin receptor has been observed in cancer tissues compared to normal tissues, particularly in cancers with an aggressive phenotype or drug resistance [64–66]. Moreover, leptin stimulates cancer cell migration, invasion, CAF induction, and CAF-mediated tumor progression [67], and changes in the polarity of tumor cells [68]. The presence of leptin also stimulates leptin receptor expression in cancer cells [69].

Leptin secretion is higher in beige than in white adipocytes [105]. In white adipocytes, the mRNA production of the leptin gene is approximately twofold higher in subcutaneous adipocytes than in the visceral adipocytes of the major omentum [70].

A Phase 3 clinical trial has reported a correlation between the efficacy of VEGFR inhibitors and blood leptin levels in patients with colorectal cancer [71]. In a preclinical study, PDX prostate cancer growth inhibition has been reported in response to leptin receptor antagonist administration [129]. Leptin may also play a role in hormone therapy resistance, as leptin levels in the blood increase due to hormone therapy for breast cancer [130]. Moreover, a randomized controlled trial reported that aerobic exercise reduced leptin levels and the risk of breast cancer in a dose-dependent manner [30].

### Resistin

Resistin is a member of the adipokine family, is an adipocyte-secreted factor whose levels increase with obesity [131]. Resistin induces insulin resistance by inhibiting AMPK phosphorylation [132]. In humans, resistin is primarily secreted by peripheral blood mononuclear cells and other organs, such as pancreatic islet cells [132, 133], whereas in rodents, its main source is adipocytes and other tissues [134]. The structure and function of human resistin also differ from those of murine resistin [132]. A strong correlation between serum resistin levels and insulin resistance has been observed in rodent studies; however, the correlation between serum resistin levels and insulin resistance in human studies is controversial [132, 135, 136]. Human resistin levels correlate strongly with visceral obesity [137]. Single nucleotide polymorphisms in human *RETN* are associated with altered plasma resistin levels, dyslipidemia, and insulin resistance, particularly in East Asian populations [138]. Animal studies using transgenic mice have reported that obesity strongly suppresses resistin secretion [139]. However, resistin secretion can be controlled by some antidiabetic drugs [139]. Resistin is also reported to be secreted from brown fat, and some antidiabetic drugs increase its secretion [78]. Animal studies in rats have reported that resistin is secreted at higher levels by females than by males and that it is secreted in the stomach, intestinal tract, skeletal muscle, and adipose tissue [79].

CAP1, decorin, ROR1, and TLR4 have been identified as resistin receptors that activate different signaling cascades [80–83]. Resistin promotes cancer proliferation via AKT and STAT signaling [84, 85], angiogenesis via VEGFR, SAPK/JNK, and NFKB signaling [86, 87], epithelial-mesenchymal transition via the WNT/β-catenin pathway [88], and invasion and metastasis via the WNT/β-catenin, TGFβR, MAPK, pathways [140]. In an animal study, the administration of resistin promoted ovarian tumor growth by regulating micro RNA (miRNA) expression [88].

In a clinical study on postoperative weight loss in patients with breast cancer, weight loss significantly decreased blood resistin levels, but did not significantly affect blood inflammatory cytokines or lipid composition [141]. In in vitro experiments, resistin has been implicated in cancer resistance and an increase in cancer stem cells [85], and has been suggested to be a potential important target for cancer therapy.

### Visfatin

Visfatin/nicotinamide phosphoribosyltransferase (NAMPT) is another member of the adipokine family of proteins [142]. Visfatin is a pre-B-cell colony-enhancing factor that promotes the maturation of early B-lineage precursor cells [143]. It is secreted by the visceral and subcutaneous adipocytes [144, 145]. A study using animal tissues reported that visfatin is more abundant in brown adipose tissues than in other types of adipose tissue, whereas in humans, brown and white adipose tissues showed no significant differences in visfatin expression [146]. Visfatin is also secreted by the liver, skeletal muscles, neutrophils, and fetal membranes [147]. Serum visfatin levels are associated with obesity, inflammation, cardiovascular diseases, and endothelial cell dysfunction [89, 90].

Hypersecretion of visfatin is correlated with worse prognosis in breast, endometrial, and renal cell cancers [91–93]. Visfatin promotes cell proliferation in endometrial cancer [94], invasiveness of liver cancer [95], and inhibition of apoptosis in breast cancer by activating the PI3K/AKT, MAPK, and ERK1/2 signaling pathways [94]. In an in vivo study in mice, visfatin promoted endometrial tumor growth by stimulating PI3K/AKT and other signaling pathways [94].

Clinical studies have reported significantly lower blood visfatin levels in pediatric patients with leukemia in complete remission [148]. In in vitro experiments, non-small cell lung cancer cell lines that became resistant to cisplatin treatment were reported to have elevated visfatin levels, whereas visfatin KO restored sensitivity to cisplatin [149]. Thus, visfatin inhibitors may contribute to increased drug resistance in patients with lung cancer.

### Chemerin

Chemerin is an adipokine that was identified in 1997 [150]. Chemerin is found in the serum, plasma, adipocytes, and the liver [151]. Chemerin, produced mainly by adipocytes and the liver, is a ligand for chemokine-like receptor 1 (CMKLR1), G-protein-coupled receptor 1, and C–C motif chemokine receptor-like 2 [152]. Chemerin has been reported to be expressed during differentiation into brown adipocytes [153] and is abundant in mouse white adipose tissue [151]. Moreover, it promotes the differentiation of bone marrow adipocytes [41]. Inhibition of chemerin secretion by antidiabetic drugs has been reported [42].

In a cohort study of over 7,000 people, chemerin concentration was significantly associated with cancer mortality [43]. In patients with breast cancer, serum chemerin levels were significantly associated with histological grade and Ki67 expression [44]. However, the role of chemerin in tumor growth remains controversial. In an in vitro study, chemerin suppressed the proliferation of ovarian cancer cell lines and could potentially regulate INFα secretion by cancer cells [45]. In addition, chemerin suppressed the viability and invasion of breast cancer cell lines [46]. In an in vivo study, the chemerin analog CG34 significantly stimulated the growth and bioluminescence signals of colorectal cancer xenografts [47]. Monoclonal antibodies targeting chemerin led to reduced lipid storage and diminished renal cancer growth by alleviating the suppression of fatty acid oxidation and ferroptosis induced by chemerin [48]. Furthermore, chemerin overexpression in breast cancer reduced tumor growth by recruiting natural killer cells and T cells in vivo [49]. In addition, chemerin overexpression suppressed hepatocellular carcinoma cell proliferation and tumor metastasis by reducing AKT phosphorylation [154]. In an in vivo study in mice, intraperitoneal chemerin administration suppressed breast tumor growth [46]. In contrast, chemerin has been reported to promote chemotaxis and migration of cutaneous squamous cell carcinoma [155], and its attenuation inhibited renal tumor growth in vivo [48]. These results suggested that chemerin may regulate different functions in cancers derived from different organs.

Currently, no randomized clinical trials of chemerin or its inhibitors in cancer have been reported in PubMed. However, an in vivo study has reported that chemerin inhibitors promoted cancer cell senescence and enhanced the therapeutic effect of cisplatin [156]. Small molecules that selectively inhibit the chemerin receptor CMKLR1 have also been reported to inhibit endometriosis growth [157], and chemerin signaling inhibitors are expected to serve as orthologous cancer therapeutics.

### Apelin

In 1998, apelin was identified as a ligand of the human APJ receptor [158]. In addition to adipocytes, apelin is broadly expressed in many organs and tissues, including the brain, kidneys, and heart [159]. Activation of apelin signaling promotes brown adipocyte differentiation [160]. Apelin further promotes the browning of white fat [161]. Apelin also promotes angiogenesis [162]. In addition, apelin signaling stimulates nitric oxide release, which promotes vasodilation by relaxing the smooth muscle cells of the arterial walls [35]. In cancer, activation of the apelin-AJP pathway promotes the peritoneal dissemination of ovarian cancer cells [36]. Moreover, the loss of apelin blocks angiogenesis in lung cancer and melanoma cells in vivo [37]. Apelin activates the PI3K/AKT pathway and promotes the proliferation, migration, and glucose uptake of pancreatic cancer cell lines [38]. Apelin promotes tumor growth by facilitating endothelial cell migration, resulting in rapid angiogenesis [39]. Apelin KO mice bearing breast cancer tumors show prolonged survival, with or without anti-angiogenic treatment [40]. In melanoma, APJ-KO suppressed angiogenesis in vivo [163]. These results suggest that apelin strongly affects the vascular environment surrounding tumors and that it is a novel cancer treatment target.

Clinical trials using apelin and apelin inhibitors have not been reported on PubMed to date, but apelin receptor expression has been reported to correlate with prognosis in patients with gastric cancer who were treated with chemoradiotherapy [164]. Moreover, in vitro and in vivo experiments using colon and prostate cancer cell lines have reported that high apelin expression alters the vascular structure and immune environment, resulting in a reduction in tumor size [165]. In contrast, tumor growth, angiogenesis, and metastasis have been reported to be suppressed in vivo in tumors in mouse models in which apelin expression was suppressed [40]. Further, ML221, an antagonist of the apelin receptor, significantly suppressed liver metastasis in breast cancer when combined with dendritic cell vaccine therapy in vivo [166]. In summary, although the function of apelin in cancer is complex, it may serve as a potential therapeutic target.

### Omentin

In 2001, omentin (also known as intelectin-1) was identified as a human lectin that binds to galactofuranosyl residues [72]. Omentin is composed of 295 amino acids and is expressed in the heart, intestine, thymus, and adipocytes [72, 73]. Omentin is expressed in large amounts in visceral white adipocytes and to some extent in subcutaneous white adipocytes [73]. Omentin is also widely expressed in organs other than adipose tissue, including the heart, intestinal tract, and kidneys [74]. Omentin levels are inversely associated with obesity and type 2 diabetes mellitus [75]. However, omentin receptors have not yet been identified.

The effect of omentin on cancer progression is controversial because the relationship between the omentin serum levels and cancer progression differs depending on the primary cancer sites and the patient’s health status [76, 77]. For example, serum omentin levels were positively correlated with colon cancer risk in study participants with a body mass index (BMI) < 30 kg/m^2^, but the correlation was not observed in participants with BMI ≥ 30 kg/m^2^ [167]. While omentin has been reported to promote apoptosis in hepatocellular carcinoma cells [168], it has also been reported to induce cell proliferation [169].

In patients with endometrial cancer, the blood levels of omentin correlate with lymph node metastasis [170], suggesting that it may be involved in the control of cancer malignancy. At present, no clinical trials targeting omentin have been conducted.

Taken together, these data suggest that omentin may have the potential to suppress tumors, but this effect is limited to certain conditions.

### miRNAs, chemokines, extracellular vesicles, and other factors

Various types of miRNAs are secreted from adipose tissues [171–173]. Studies using the 3T3-L1 cell line reported that adipocytes secrete a large number of extracellular vesicles [174] and that a large number of miRNAs are contained within these extracellular vesicles [175]. In addition, specific miRNAs appear to be expressed in different adipocyte types [174].

Adipose tissue secretes various chemokines, such as CCL2, CCL20, and CXCL5, at regular intervals or during cancer therapy [176, 177]. Consequently, they induce inflammation and the reorganization of adipose tissue, which affects the progression of cancer.

Moreover, adipocytes also secrete various other factors, including free fatty acids [178, 179], lipokines [180, 181], vasoactive proteins [182, 183], and matrix metalloproteinases [184, 185], which are directly or indirectly involved in cancer nutrition, growth, and metastasis.

## Conclusions

Aging and metabolic syndrome are both recognized in developed countries, and as a result, the total number of cancer patients with organs with adipocyte accumulation and adipocytes replacement is expected to increase [186–189]. Cancer therapy targeting adipocytes has the potential to be an innovative treatment not only for metastatic cancer, but also for a wide range of cancer patients across organs. In this review, we described the effects of various adipokines and other adipocyte-secreted factors on cancer. The molecular mechanisms by which the factors secreted by adipocytes affect cancer and the resulting effects on cancer survival, proliferation, invasion, metastasis, and resistance to therapy are diverse. Notably, while adipokines mainly promote tumor growth, certain adipokines, such as adiponectin and chemerin, have the potential to suppress tumor growth. Therefore, it is essential to consider the overall balance of adipocyte-derived factors to understand the role of adipocytes on tumors. We propose the establishment and expansion of “adipo-oncology” as a research field to enhance the comprehensive understanding of the role of adipocytes in metastatic cancers and the development of more robust metastatic cancer treatments. Integrating information of mechanisms regulating cancer by adipocyte-secreted factors, understanding the secretion status of each secreted factor, the type and distribution of adipocytes in patients with cancer, and carefully controlling fat secretion factors in each patient may lead to useful cancer treatments. Therefore, it is necessary to accumulate basic and clinical data for the future development of novel cancer therapies.
